# Recovery of virus-free Almond (*Prunus dulcis*) cultivars by somatic embryogenesis from meristem undergone thermotherapy

**DOI:** 10.1038/s41598-022-19269-3

**Published:** 2022-09-02

**Authors:** Maryam Ebrahimi, Ali Akbar Habashi, Masoumeh Emadpour, Nooshin Kazemi

**Affiliations:** 1 Department of Agricultural Biotechnology, Tarbiat Modares University, Tehran, Iran; 2 Agricultural Biotechnology Research Institute of Iran (ABRII), Karaj, Iran; 3 Temperate Fruits Research Center, Horticultural Sciences Research Institute, Agricultural Research, Education and Extension Organization (AREEO), Karaj, Iran

**Keywords:** Biological techniques, Plant sciences

## Abstract

One of the world's main horticulture problems is the contamination of fruit trees with a variety of plant diseases, especially viral and pseudo-viral diseases. Due to the non-sexual propagation of the trees, these diseases have been transmitted to different parts of the world. The main aim of this study was to obtain a new effective method for virus elimination from almond cultivars, which was performed in two phases. In the first phase, we tested various almond cultivars with ELISA and RT-PCR. The results showed the infection of mother plantlets. So, three types of in vitro thermotherapy treatments were performed on infected plants to make them virus-free. The plantlets obtained from 0.5 mm meristem treated with the first type of thermotherapy (TH1: 8 h at 27 °C and 16 h at 38 °C for 18 days) showed the highest percentage of elimination of ApM, ACLS and TRS viruses. In the second phase, meristems were cultured on MS medium containing 0, 0.5, 1 and 2 mg/L 2,4-D with 1 mg/L TDZ and after two weeks, thermotherapy treatments were performed. The results showed, combining three methods of thermotherapy (TH1), meristem culture and somatic embryogenesis induction from meristem on MS medium supplemented with 0.5 mg/L 2,4-D and 1 mg/L TDZ is the most effective and safe technique for virus eradication without meristem size challenges. The samples that were diagnosed as virus-free were proliferated in temporary immersion bioreactor systems, and rooted to be used for later propagation and establishment of mother healthy orchards.

## Introduction

Almonds are one of the most important nuts in the world. With the increase in almond cultivation around the world, scientists are looking for factors that increase its yield. Therefore, the implementation of research programs in the field of health of commercial cultivars in each country is of great importance and leads to an increase in production per unit area. On the other hand, the yield in orchards with healthy cultivars is typically about 1.5–2 times more than control orchards^1^.

However, it is important to have knowledge about the viral contaminants in almond trees, and the need to provide healthy vegetative materials for mother orchards. Various viruses have been reported in almonds, including prunus necrotic ringspot virus (PNRSV), apple chlorotic leaf spot virus (ACLSV), apple mosaic virus (ApMV), tomato ringspot virus (TRSV) and Mediterranean almond viruses^2^. Therefore, the use of efficient techniques such as shoot tip meristem culture, somatic embryogenesis and thermotherapy can be used to obtain virus-free plants^3–7^. Meristems are virus-free in most cases due to the lack of a vascular system. Studies have shown that culturing meristems of smaller sizes successfully results in virus-free plants^8–12^. In addition, high temperatures prevent virus replication by inhibiting virus transcription enzymes and reduce the transportation of virus towards the apical meristem^13^. Somatic embryogenesis eliminates the virus due to the lack of connection between the somatic embryo and the mother plant. In addition, somatic embryos are induced from non-vascular tissues, while viruses are mostly confined to vascular tissue, therefore, the chances of eliminating the virus increase^14^.

Finding a proper technique in order to eradicate virus from almond trees is an important key to achieving healthy orchards and crops. Therefore, in the first phase of the present study, the effectiveness of thermotherapy treatments with meristem culture and in the second phase, for the first time, induction of somatic embryogenesis from meristem undergone thermotherapy was studied to investigate the role of these treatments in viral almond eradication. The presented results could develop the culture of virus-free germplasm of these trees as well as other fruit trees.

## Materials and methods

### Explant preparations and general procedures of in vitro proliferation

Shoot samples were prepared from six local and commercial almond cultivars including Shahroodi, Shokofeh, Araz, Scandar, Nonpareil and Sahand from the almond germplasm collection of Horticultural Sciences Research Institute (Karaj, Iran). The single lateral buds were transferred to the (MS)^15^ containing 1 mg/L BAP, 0.5 mg/L GA3, 0.01 mg/L IBA and 30 g/l sucrose and 7 g/L plant-agar after disinfection with 70% alcohol for 1 min, then with sodium hypochlorite (2.5%) treatment for 10 min and nano-Silver (2.5%) for 10 min.

Different basal media including MS, woody plant medium (WPM)^16^ and (QL)^17^ and three types of growth regulators combination were studied to optimize the proliferation medium as follows (Table 1):

**Table 1:** The MS, WPM, QL media with growth regulator combinations for almond explants proliferation.

Combinations	Growth regulators (mg/L)			
	BAP	IBA	GA3	TDZ
1^a^	1	0.01	0.5	0
2	1	0.01	0	0
3	0.01	0.5	0	0.5

^a^This combination was also investigated with the temporary immersion bioreactor system.

Each medium contained 30 g/L sucrose and 7 g/L plant-agar. After pH adjustment on 5.8, the media were autoclaved for 15 min at 121 °C (1.2 kPa) and then the explants obtained from the establishment stage were cultured. The experiment was performed as factorial arrangement in completely randomized design with 4 replications. In a separate experiment, the best plant and the best medium were selected and the performance of the culture medium was evaluated as liquid (culturing in temporary immersion bioreactor system) and solid. Hence, the explants grown on MS medium with 1 mg/L BAP were sub-cultured to bioreactor container containing 330 ml of liquid MS medium supplemented with 1 mg/L BAP, 0.5 mg/L GA3, 0.01 mg/L IBA and 3% sucrose. Four explants with 1.5 – 2 cm length from lateral buds were cultured in each bioreactor container. The flow of the culture medium from the reservoir to the explants was regulated once every 24 h for 10 min. Cultures were incubated at a temperature of 25 ± 2 °C and 16 h light photoperiod at about 3000 lx. The solid medium was prepared with the same compounds as the liquid medium supplemented with 7 g/L plant-agar. Four explants with 1.5–2 cm length from lateral buds were cultured in each jar container. After 30 days, fresh weight (FW), dry weight (DW), shoot number (SN) and shoot length (SL) were measured. The experiment was performed in completely randomized design. The treatments were compared in 3 replications (3 jars or 3 bioreactor containers), which were incubated at a temperature of 25 ± 2 °C and 16 h light photoperiod at about 3000 lx.

### Virus detection using reverse transcriptase-PCR (RT-PCR)

Before applying virus eradication treatments, in vitro cultured almond cultivars was evaluated by ELISA kit (Bioreba, Reinach, Switzerland) for the presence of TRS virus. The plantlets were also tested for this virus by reverse transcription polymerase chain reaction (RT-PCR) assays. Total RNA was extracted from almond samples for RT-PCR and then synthesis of cDNA by reverse transcription and subsequent PCR was done by using the two-step kit (Thermo Scientific, Switzerland).

Before applying virus eradication treatments, we examined the contamination of mother plants using RT-PCR. The evaluated viruses included TRS, ACLS, and ApM. A proper sequence was selected to design primer pairs for each of the examined viruses from the previous reports (Table 2).

**Table 2:** The sequence of primer pairs used to amplify a specific sequence in cDNA of each examined virus.

Virus name	Product size (bp)	Primer sequence	References
ACLSV	677	F:TTCATGGAAAGACAGGGGCAA R:AAGTCTACAGGCTATTTATTATAAGTCTAA	^18^
ApMV	450	F:CGTAGAGGAGGACAGCTTGG R:CCGGTGGTAACTCACTCGTT	^19^
TRSV	315	F:CAGGGGCGTGAGTGGGGGCTC R:CAATACGGTAAGTGCACACCCG	^20^
Rubc1	184	F:TACTTGAACGCTACTGCAG R:CTGCATGCATTGCACGGTG	^21^

Primers to amplify part of a Rubisco subunit cDNA used as control.

For specific amplification of the desired sequence, different annealing temperatures of the primer pairs were evaluated using temperature gradient. The temperature of 59 °C was finally used for all primer pairs. The small PCR products were loaded on the 1.5% agarose gels for detection.

Extraction of RNA from plant leaves was performed according to the modified CTAB protocol^22^. After completing the extraction process, concentration and quality of the extracted RNA were checked using a NanoDrop device and then by electrophoresis. Subsequently, about 4 µL of each extracted RNA sample was loaded on 1.5% agarose gel and after electrophoresis the resulting bands were evaluated. Based on the concentration (ng/µL) obtained from NanoDrop, the amount of the extracted compound containing 1 µg of RNA was taken and l µL Dnase enzyme plus l µL buffer was used to eliminate DNA.

The successful synthesis of cDNA was examined with a proper primer pair from the housekeeping gene (a Rubisco subunit) by polymerase chain reaction. After observing the expected PCR product band on the electrophoresis gel, the manufactured cDNA was stored for later use in − 20 °C.

After ensuring that the cDNA was constructed, the PCR reaction was performed using all virus specific primers for each sample of the constructed cDNA from mother plants. The PCR reaction product of each sample was loaded on the agarose gel and separated by electrophoresis. These diagnostic tests were also performed after the virus eradication operation to determine healthy plants. After virus-free operations and plantlets propagation, the percentage of virus-free plantlets detected by RT-PCR for each type of treatment was evaluated in a factorial arrangement in a completely randomized design with 3 replications.

### Virus elimination by thermotherapy and shoot tip meristem culture

Two experiments were designed to optimize virus elimination by thermotherapy and shoot tip meristem culture. In the first experiment, three thermotherapy methods were applied on proliferated plantlets including TH1: 8 h at 27 °C and 16 h at 38 °C for 18 days, TH2: 10 days at 38 °C and TH3: 11 days at 38 °C; for gradual adaptation to this temperature, explants were first placed at a lower temperature (27 °C) and temperature was gradually increased (by around 3 °C increase per day). The experiment was performed as factorial arrangement in completely randomized design with 3 replications (petri dish) containing four 0.5 mm meristems. Meristem culturing was performed after thermotherapy (modified MS medium containing 0.4 mg/L BAP, 0.1 mg/L IBA, 30 g/L sucrose and 7 g/L plant-agar). In the second experiment, meristems were separated from the explants undergone TH2. The primary separated apical meristem was approximately 5 mm with the lateral leaflets. Finally, the leaflets were removed and 0.5 and 1 mm meristems were cultured on the modified MS medium supplemented with 30 g/L sucrose, 7 g/L plant-agar, 0.1, 0.2 and 0.4 mg/L BAP and 0.1 mg/L IBA. The explants were incubated in a 24 ± 2 °C growth rooms with a 16 h light and 8 h of darkness photoperiod and light intensity of 2000 lx. There were 4 repetitions (petri dish) for each treatment; containing four meristems and the experiment was performed as factorial arrangement in completely randomized design. Following 30–35 days, the grown meristems were transferred to a new culture medium, and after sufficient growth, the virus tracking tests were applied.

### Virus eradication by induction of somatic embryogenesis from meristem undergone thermotherapy

Meristems were cut to 0.5 and 1 mm sizes and cultured on the modified MS medium supplemented with 0, 0.5, 1 and 2 mg/L 2,4-Dichlorofenoxiacetic Acid (2,4-D), 1 mg/L thidiazuron (TDZ), 30 g/L sucrose and 7 g/L plant-agar. After two weeks of incubating at 24 ± 2 °C in a dark environment, they underwent two methods of thermotherapy (TH1: 18 days at 27 °C for 8 h and 38 °C for 16 h per day, TH2: 10 days at 38 °C) in a dark environment; for gradual adaptation to this temperature, explants were first placed at a lower temperature (27 °C) and temperature was gradually increased (by around 3 °C increase per day). There were three repetitions (petri dish) for each treatment, containing four meristems and the experiment was performed as factorial arrangement in completely randomized design. After thermotherapy, developed plantlets were propagated in temporary immersion bioreactor system containing liquid MS medium supplemented with 1 mg/L BAP, 0.5 mg/L GA3, 0.01 mg/L IBA and 3% sucrose.

### Propagation of virus-free almond plants

The explants were examined for ACLS, ApM and TRS viruses using molecular RT-PCR method. In addition, the serological ELISA method was carried out for TRS virus detection. Virus-free explants were amplified in temporary immersion bioreactor system supplemented with 1 mg/L BAP, 0.5 mg/L GA3, 0.01 mg/L IBA and 3% sucrose and then rooted in the half strength MS (1/2MS) medium supplemented with 1 mg/L IBA and 0.5 mg/L IAA. Proliferated shoots of 3 to 5 cm were used for rooting on MS or 1/2MS media supplemented by different concentrations of Indole-3-butyric acid (IBA) and Indole-3-acetic acid (IAA). The experiment was performed as factorial arrangement in a completely randomized design with 6 treatments and 4 replications. Explants were cultured in jam jars, with 3 explants in each, and were transferred to the photoperiod of 16 h light and 8 h darkness. Root number (RN) and root length (RL) were recorded after 6 weeks. Rooting treatments included:

1. Plant growth regulator free MS
2. Plant growth regulator free 1/2MS
3. MS medium + IAA (1 mg/L)
4. 1/2MS medium + IAA (1 mg/L)
5. MS medium + IAA (1 mg/L) + IBA (0.5 mg/L)
6. 1/2MS medium + IAA (1 mg/L) + IBA (0.5 mg/L)

### Statistical analysis

In the present study, as mentioned in the previous sections, seven experiments were performed, one in a completely randomized design and the other six in factorial arrangements in a completely randomized design. This study was done on 6 cultivars. The results of the interaction effects of 6 cultivars × different factors for each experiment are presented in the supplemental file and the interaction effects of 2 cultivars × different factors are presented as the main results. Statistical analysis of the measured characteristics was conducted using SAS (Version 9). Multivariate variance analysis was applied to determine the significance of differences between assays. Comparative mean LSD test (*p* < 0.05 or *p* < 0.01) was applied to establish the significance of the differences between each group, and the relevant graphs were drawn using Microsoft Excel. All data is displayed with standard error (± S.E).

### Statement

All experiments were performed in accordance with relevant guidelines and regulations.

## Results

### Optimization of proliferation medium

To determine the optimum medium for proliferation from six cultivars of almond, the shoot number and shoot length of the explants were investigated using combinations of various hormonal treatments (BAP, IBA, and GA3) with different types of media (MS, QL, and WPM). The results indicated that average number of shoots per explant for the 6 cultivars was 3.15, 1, and 0.78 on MS, QL, and WPM, respectively (Supplementary Fig. S1a). Indeed, explants cultured on MS medium produced significantly more shoots than those on the other two media. The highest number of shoots per explant for the cultivars was obtained on MS medium supplemented with 1 mg/L BAP, 0.01 mg/L IBA and 0.5 mg/L GA3 (hormonal combination 1). On this medium, Shokofeh and Araz explants produced 5 and 3.25 average number of shoots per explant, respectively (Table 3). Culturing the explants on various media showed different results in terms of shoot length. In fact, the 6 cultivars grown on MS, QL, and WPM generated averages of tallest shoot length of 2.66, 0.88, and 0.61 cm, respectively, showing significantly more enhanced results on MS medium than others (Supplementary Fig. S1b). The highest shoot length per explant for the cultivars was also obtained on MS medium supplemented with hormonal combination 1. Culturing on this medium, Shokofeh and Araz explants generated 5.25 and 3 cm as average length of the tallest shoot per explant, respectively (Table 3).

**Table 3:** Interaction effects of 2 cultivars × 3 media × 3 hormonal combinations on average of shoot number and tallest shoot length, after 4 weeks.

Combinations	Average of shoot number		Average of tallest shoot length (cm)	
	Shokofeh	Araz	Shokofeh	Araz
MS1	5 ± 0 a	3.25 ± 0.25 d	5.25 ± 0.12 a	3 ± 0 c
MS2	4 ± 0 b	1 ± 0 g	2.72 ± 0.10 cd	1.5 ± 0 g
MS3	1 ± 0 g	2 ± 0 f.	0.52 ± 0 h	1.5 ± 0 g
QL1	1 ± 0 g	2 ± 0 f.	0.5 ± 0 h	2 ± 0 f.
QL2	1 ± 0 g	0 h	1.49 ± 0.06 g	0 i
QL3	3.75 ± 0.25 c	0 h	2.62 ± 0.07 de	0 i
WPM1	2.91 ± 0.8 e	0 h	3.54 ± 0.38 b	0 i
WPM2	3 ± 0 e	0 h	2.55 ± 0.12 de	0 i
WPM3	4 ± 0 b	0 h	2.37 ± 0.14 e	0 i

**Figure 1:**
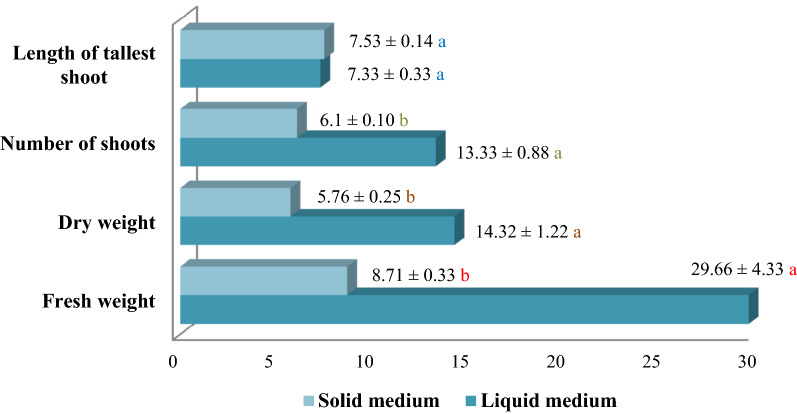
Comparison of in vitro growth indices of almond (shahroodi) in solid and liquid MS medium containing 1 mg/L BAP + 0.01 mg/L IBA + 0.5 mg/L GA3. (n = 3, *p* < 0.01, ± S.E).

**Figure 2:**
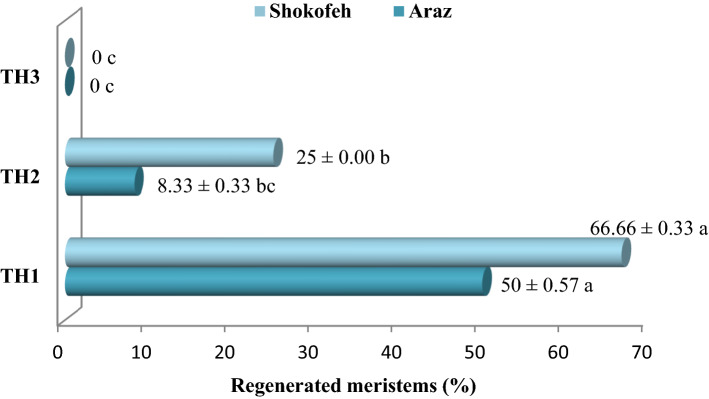
Interaction effects of 2 cultivars × 3 thermotherapy treatments on the percentage of regenerated meristems; TH1: 18 days at 27 °C for 8 h and 38 °C for 16 h, TH2: 10 days at 38 °C and TH3: 11 days at 38 °C. (n = 3, *p* < 0.05, ± S.E).

**Figure 3:**
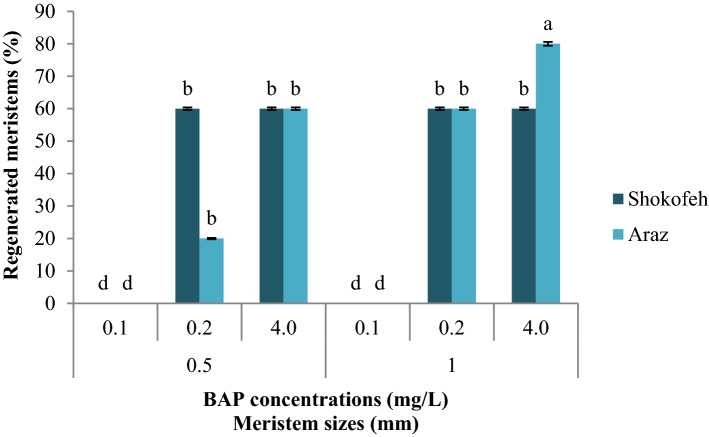
Interaction effects of 2 cultivars × 2 sizes of meristem × 3 hormonal combinations on the percentage of regenerated meristems. (n = 4, *p* < 0.01, ± S.E).

The Figures 1, 2 and 3 on the side of each medium mean the hormonal combination 1, 2 and 3; (1) 1 mg/L BAP + 0.01 mg/L IBA + 0.5 mg/L GA_3,_ (2) 1 mg/L BAP + 0.01 mg/L IBA and (3) 0.01 mg/L IBA + 0.5 mg/L BAP + 0.5 mg/L thidiazuron (TDZ). (n = 4, *p* < 0.01, ± S.E).

The results of this study showed that temporary immersion bioreactor system is a suitable system for micropropagation of almonds (Shahroodi cultivar). According to the results of this study, number of shoots, fresh weight and dry weight as growth indices, in temporary immersion bioreactor system have increased significantly compared to solid culture medium (Supplementary Figs. S2 and S3). According to the Fig. 1, fresh weight and dry weight (8.71 and 5.76 g, respectively) of shoots on solid medium compared to bioreactor system (29.66 and 14.32 g, respectively) were much lower. In this regard, it can be stated that this advantage of the temporary immersion bioreactor system is related to the better availability and absorbability of the elements by the explants and the lack of stabilization of the elements in the gel.

### Optimization of rooting culture medium

As shown in Table 4 and Supplementary Fig. S4, the average of root number and average of tallest root length were related to ½MS medium with a combination of two hormones including IAA (1 mg/L) and IBA (0.5 mg/L) for all six cultivars. Shokofeh and Araz had 4.5 and 4 roots per explant and in the case of the average length of the tallest roots; it was 16.5 and 14.5 cm, respectively (Table 4).

**Table 4:** Interaction effects of 2 cultivars × 2 media × 3 hormonal combinations on average of root number and average of tallest root length.

Media and hormonal combinations	Average of root number		Average of tallest root length (cm)	
	Shokofeh	Araz	Shokofeh	Araz
MS1	0 d	0 d	0 e	0 e
MS2	0 d	0 d	0 e	0 e
MS3	3 ± 0 c	3.37 ± 0.12 c	12.37 ± 0.23 c	10.25 ± 0.25 d
1/2MS1	0 d	0 d	0 e	0 e
1/2MS2	0 d	0 d	0 e	0 e
1/2MS3	4.5 ± 0.28 a	4 ± 0.40 b	16.25 ± 0.25 d	14.5 ± 0.28 b

The Figures 1, 2 and 3 on the side of each medium mean the hormonal combinations 1, 2 and 3; (1) Plant growth regulator free medium, (2) IAA (1 mg/L) and (3) IAA (1 mg/L) + IBA (0.5 mg/L). (n = 4, *p* < 0.01, ± S.E).

### The results of molecular sample monitoring

The results of RNA extraction using CTAB showed high concentration and low contamination. The extracted RNA concentration was assayed with a NanoDrop and the result of the RNA electrophoresis on the 1.5% agarose gel was performed. Then, after adding Dnase and checking DNA deletion using Rubisco internal gene, high quality RNAs were used to make cDNA.

After ensuring the cDNA synthesis and quality, the PCR reaction was performed with specific primers of the ACLS, ApM and TRS virus coat proteins for all cDNA samples. The results obtained from this section were as follows.

RT-PCR results showed that the explants of Shahroodi and Shokofeh were infected with ACLS virus but the explants of Araz, Sahand, Nonpareil and Scandar were not infected with this virus. The results of examination of explants with specific primer of ApM virus showed that Sahand, Shokofeh and Nonpareil were infected with ApM virus. Araz, Sahand and Scandar were infected with TRS virus, as well. These experiments were repeated three times to confirm the results (Supplementary Fig. S5).

### The effects of virus eliminating treatments on explants growth and Surviving

As shown in Fig. 2, TH1: 18 days at 27 °C for 8 h and 38 °C for 16 h was more effective on the percentage of regenerated meristems and, in addition, this treatment was able to produce new shoots, but TH1 and TH2 treatments were selected to be compared in virus-free performance in subsequent experiments. Interaction effects of 6 cultivars × 3 types of thermotherapy treatment on percentage of regenerated meristems showed the same results (Supplementary Fig. S6).

Based on the obtained results, for Shokofeh cultivar, both media containing 0.2 and 0.4 mg/L BAP were successful and there was no significant difference between 0.5 and 1 mm for meristem regeneration. Araz cultivar showed the highest number of meristem regenerated in medium containing 0.4 mg/L BAP and 1 mm meristem. There was no significant difference between the treatment containing 0.4 mg/L BAP and 0.5 mm meristem compared to the treatment containing 0.2 mg/L BAP and 1 mm meristem (Fig. 3). Based on the above results and the result of the other 4 cultivars (Supplementary Table S1), MS culture medium containing 0.4 mg/L BAP and 0.1 mg/L IBA was selected as the medium with the highest percentage of regenerated meristems (Supplementary Fig. S7) and 0.5 and 1 mm meristems were selected to be compared in virus-free performance in subsequent experiments.

### Evaluation of ACLS, ApM and TRS virus infection of explants with virus-specific primers

As reported in the previous section, Shahroodi and Shokofeh cultivars were infected with ACLS virus. The results of Table 5 and Supplementary Table S2 show that 0.5 mm meristem undergone TH1 treatment resulted in the highest percentage of virus free plantlets so that Shokofeh cultivar was 78% virus-free, and plantlets obtained from 1 mm meristem, which were treated with thermotherapy similarly, had no significant difference in percentage of obtained ACLS virus-free plantlets with the ones obtained from 0.5 mm meristem treated with TH2. The lowest percentage of ACLS virus elimination was found in plantlets obtained from 1 mm meristem treated with TH2, which was 25.33% for Shokofeh cultivar. According to the results of the previous section, Shokofeh cultivar was infected with ApM virus, too. Plantlets obtained from 0.5 mm meristem undergone TH1 treatment had the highest percentage of ApM virus elimination, which was 68.33%. The lowest percentage of ApM virus elimination was 21%, obtained from 1 mm meristem undergone TH2 treatment. According to the results of the previous section, Araz cultivar was infected with TRS virus and showed the highest percentage of virus elimination in plantlets obtained from 0.5 mm meristem, which were treated with TH1 (61.66%), and the lowest percentage of virus-free plantlets was obtained from 1 mm meristem undergone TH2 treatment (18.66%). In general, the results showed that 0.5 mm meristem treated with TH1 were more effective than other treatments for ACLS, ApM and TRS virus elimination percentage (Table 5 and Supplementary Table S2).

**Table 5:**
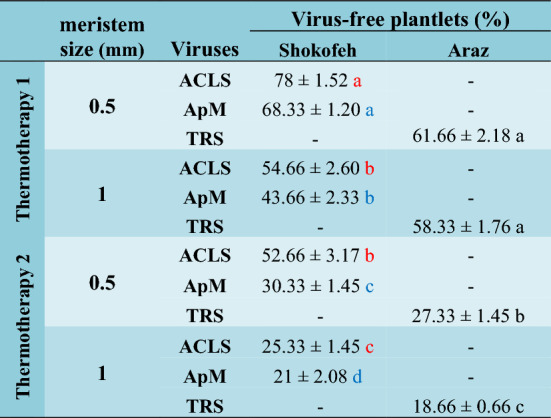
Interaction effects of each of 2 cultivars × 2 sizes of meristem × 2 thermotherapy treatments on the percentage of virus elimination. (n = 3, *p* < 0.01, ± S.E).

### Detection of TRS virus through ELISA and comparison of results obtained by ELISA and RT-PCR

The results obtained by RT-PCR method showed that three cultivars Araz, Sahand, Scandar were infected with this virus, but the results of ELISA showed that only Araz and Sahand cultivars were infected with this virus and it was not possible to comment definitively on Scandar cultivar (Supplementary Table S3). Therefore, it can be said that the RT-PCR method is a more reliable method in this regard.

### Induction of somatic embryogenesis from meristem undergone thermotherapy and RT-PCR result

The results showed that 0.5 and 1 mg/L 2,4-D with TH1 treatment were produced embryogenic calluses and plantlets, but 0.5 mg/L 2,4-D had the highest percentage of embryogenic calluses (91.66%) and there was no significant difference between the 0.5 and 1 mm meristem sizes in this respect. In terms of the percentage of plantlets produced from the determined embryogenic calluses, the highest performance was related to meristems with sizes 0.5 and 1 mm and concentration of 0.5 mg/L 2,4-D undergone TH1 for both cultivars (Table 6). Although TH2 led to the production of embryogenic calluses, no plantlets were obtained. So it is not effective (Supplementary Fig. S8).

**Table 6:** Interaction effects of 2 cultivars, meristems sizes and thermotherapy treatments on the percentage of embryogenic calluses and plantlets (n = 3, *p* < 0.01, ± S.E).

				2,4.D concentrations (mg/L)	Embryogenic calluses (%)	Plantlets (%)
Shokofeh	Thermotherapy 1	Meristem size	0.5 mm	0	0 e	0 d
				0.5	91.66 ± 0.33 a	100 ± 0.00 a
				1	41.66 ± 0.33 c	83.33 ± 0.66 b
				2	0 e	0 d
			1 mm	0	0 e	0 d
				0.5	91.66 ± 0.33 a	100 ± 0.00 a
				1	41.66 ± 0.33 c	100 ± 0.00 a
				2	0 e	0 d
	Thermotherapy 2		0.5 mm	0	0 e	0 d
				0.5	41.66 ± 0.33 c	0 d
				1	0 e	0 d
				2	0 e	0 d
			1 mm	0	0 e	0 d
				0.5	16.66 ± 0.33 d	0 d
				1	0 e	0 d
				2	0 e	0 d
Araz	Thermotherapy 1	Meristem size	0.5 mm	0	0 e	0 d
				0.5	91.66 ± 0.33 a	91.66 ± 0.33 ab
				1	33.33 ± 0.33 c	16.66 ± 0.66 c
				2	0 e	0 d
			1 mm	0	0 e	0 d
				0.5	91.66 ± 0.33 a	100 ± 0.00 a
				1	58.33 ± 0.33 b	83.33 ± 0.66 b
				2	0 e	0 d
	Thermotherapy 2		0.5 mm	0	0 e	0 d
				0.5	16.66 ± 0.33 d	0 d
				1	0 e	0 d
				2	0 e	0 d
			1 mm	0	0 e	0 d
				0.5	33.33 ± 0.33 c	0 d
				1	0 e	0 d
				2	0 e	0 d

Plantlets obtained from each treatment were propagated separately and then, by RT-PCR test, Shokofeh cultivar for ACLS and ApM viruses and Araz cultivar for TRS virus were evaluated with specific primers for each virus. The results showed that all plantlets obtained were 100% virus-free and it can be said that all the challenges related to meristem size were met in this method.

## Discussion

It is known that varied media as well as various combinations of hormonal treatments could influence proliferation and growth of almond explants^1,23–25^. Our results showed that MS medium supplemented with 1 mg/L BAP, 0.01 mg/L IBA and 0.5 mg/L GA3 has the most positive impact on shoot number and shoot length of the cultivars. In a study, the effect of different combinations of BAP and GA_3_ on rapid proliferation of nodal explants, bud sprouting rate and survival percentage in almond rootstock were investigated. The result showed that MS medium containing 0.1 mg/L BAP and 0.2 mg/L GA_3_ increased the bud sprouting^23^. In another study, almond establishment in culture medium containing 1 mg/L BAP showed the highest bud sprouting^25^. Additionally, Nonpareil cultivar showed shoot proliferation in MS medium containing 2% sucrose and 1 mg/L BAP^26^. The best shoot proliferation of Yallsinki cultivar explants was also reported in MS medium containing 30 g/L sucrose, 7 g/L plant-agar and 1 mg/L BAP^1^. Furthermore, the reports on proliferation of almond hybrid rootstocks indicated the successful use of MS and ½MS media with similar concentration of hormones as the current study^24^. All of these reports highlight the key role of appropriate concentration of BAP in almond in vitro proliferation. In addition, the use of MS culture medium has been generally a confident factor used in this regard. Therefore, the previous reports confirm the results of the current study on proliferation values of almond cultivars.

In a study^27^, The Prunus Rootpac 20® micropropagation in temporary immersion bioreactor system improved the stem length and fresh weight compared to culture in medium containing semi-solid agar. In another study, the possibility of using temporary immersion bioreactor system and optimizing tissue culture methods in GF677 micropropagation was investigated. In this study, the results showed that the proliferation rate of temporary immersion bioreactor system, number of shoots and biomass production were significantly different from in vitro solid culture. Micropropagation performance in temporary immersion bioreactor system was much more efficient than the conventional system, and plant micropropagation using temporary immersion bioreactor system could reduce production costs and make it an economical method^28^. Micropropagation through the use of temporary immersion bioreactor systems is considered as an alternative to increase the production efficiency of in vitro explants and reduce costs by automating the process. The aim of this study was to compare different micropropagation techniques of pear rootstocks and showed that in general, temporary immersion bioreactor systems are recommended for in vitro propagation of most of the studied pear rootstocks^29^. To propagate a reasonable number of plants in the shortest possible time, different in vitro systems were evaluated: solid medium and temporary immersion bioreactor with two different culture media in each system. The study showed that temporary immersion bioreactor was the best micropropagation system in pears^30^. Limited reports on culture of few numbers of species in temporary immersion bioreactor system have been published, indicating the feasibility of this technology. Therefore, previous studies confirm the results of the present study based on the superiority of culture in temporary immersion bioreactor system for micropropagation. Given these results, the use of temporary immersion bioreactor in the commercial propagation of valuable plants is so clear.

Shoots of *Prunus dulcis* Mill, Ne Plus Ultra and Nonpareil cultivars were cultured for 4 weeks at 4 °C in MS medium without growth regulators in low light conditions. Different concentrations of IBA and NAA hormones were compared to determine the optimal auxin for rooting. In addition, the effects of darkness, phluoroglucinol and basal salt composition were investigated. The results showed that darkness did not improve rooting ability while ½ MS medium had sufficient strength for rooting of Ne Plus Ultra^31^. In another study, MS medium without plant growth regulators was used to root a number of plants belonging to Prunus genus^8^.

Generally, in vitro virus eradication based on meristem culture is common and in addition, the other treatments including thermotherapy, chemotherapy, electrotherapy and cryotherapy can be used. During each of these treatments, the explants are subjected to a number of stresses that can lead to low survival rates, growth inhibition, stunted growth, or abnormal morphology. The sensitivity of genotypes to the treatments of these processes is very different, and the degradation degree largely depends on the plant physiological conditions. The exposure time to each of the processes affects the damage extent in treatments^13^. In a study, it was stated that in order to elimination the apple chlorotic leaf spot virus (ACLSV) from the infected plum tree explants, in vitro thermotherapy was performed for one week at 38 °C with meristem tip culture^3^. In another study on apple cultivars, they examined the combined thermotherapy and cryotherapy effects. In this method, alternating temperatures of 38 °C (day) and 30 °C (night) were applied. Combining 4 weeks of thermotherapy with cryotherapy resulted in a high percentage of eradication^32^. Cryotherapy is a method to remove obligatory pathogens from meristem cells, which is a relative success. On the other hand, there is a possibility of long-term cryopreservation of pathogens in this method. Therefore, cryotherapy acts as a double-edged sword and thermotherapy is preferred^33,34^. Thermotherapy for more than 25 days caused the leaves to turn brown and the shoots to die. Culture of shoot tips along with thermotherapy (37 °C for 35 days) was necessary to regenerate virus free plants^8^. Increasing temperature and duration of thermotherapy has a positive correlation with virus eradication frequency. These two parameters vary depending on the type of virus and plant species and the virus-host combination. In fact, these two parameters should be optimized in such a way that it allows for the treated plant to survive and grow while inactivating the virus. Thermotherapy has several advantages over other methods such as cryotherapy, but many plants cannot tolerate high constant temperatures. Therefore, thermotherapy using alternating day/night temperature causes the growth and survival of the treated plant as well as the eradication of the virus^34,35^. In one study, thermotherapy was performed at 37–40 °C for 4 weeks and then apple meristems of different sizes were cultured. The size of the meristems affected their survival. Meristems of 0.6–0.7 mm had the highest percentage of establishment. MS medium with BA (1 mg/L), IBA (0.05 mg/L) and GA3 (0.1 mg/L) was the best culture medium for meristem establishment^36^. In another study, sweet cherry meristem explants (less than 1 mm) were isolated from the tip of the shoot and cultured on MS medium. The results showed that there was no significant difference between cultivars in terms of survival index as well as proliferation rate and shoot length^37^. In general, the size of the shoot tip has a positive correlation with survival and successful production of plantlets and a negative correlation with the frequency of virus eradication^35^.

According to Table 5, it can be said that the RT-PCR method is a more reliable method in this regard. However, for mass-indexing of viruses, ELISA is widely used because of its speed^38^. Detecting fruit tree viruses has been challenging for a long time; there are two reasons for this: in the first case, the viral titer is often low, and in the second case, the viral is unevenly distributed among the tissues and branches of different fruit trees. RT-PCR consists of two steps involving viral RNA transcription using total extracted RNA and PCR amplification from viral cDNA^39^. The identification of plant viruses is of great importance as a prelude to successful management of a viral disease. Recent advances in molecular biology have led to the development of new, sensitive, and effective diagnostic methods. Current trends in plant virus detection tools have provided equipment that do not reduce sensitivity and reproducibility, such as RT-PCR^40^.

Somatic embryogenesis induction was performed from different tissues of *Prunus dulcis*. For example, cultivation of anthers in P medium, cultivation of different parts of immature cotyledons (proximal, median and distal) on MS medium containing 0.2 mg/L BAP and 0.01 mg/L IBA with different concentrations of TDZ (1, 2, 3 and 4 mg/L) plays an important role in initiating the induction of somatic embryogenesis in the proximal and median parts of the cotyledons^41,42^. In another species of Prunus (*P. cerasus*), immature cotyledons were isolated after pollination and cultured on MS medium and somatic embryogenesis was reported to occur mainly when using 2,4-D in addition to kinetin^43^.

Somatic embryogenesis is performed to produce virus-free plantlets from different plant tissues, for example, somatic embryogenesis from anthers was the most promising approach to producing arabis mosaic virus-free grape plants^44^. Also, virus-free garlic plants were regenerated through somatic embryogenesis from basal parts of cloves of two Croatian garlic ecotypes on MS medium containing 0.1 mg/L 2,4-D. The results showed that the elimination of onion yellow dwarf virus (OYDV), leek yellow stripe virus (LYSV) and garlic common latent virus (GCLV) was as successful as other methods^45^. Somatic embryogenesis of tepal flower tissue in *Hippeastrum hybridum* was performed on MS medium containing 0.5 mg/L 2,4-D and 1 mg/L TDZ. Plantlets from somatic embryos were successfully free of Cucumber mosaic virus and Hippeastrum mosaic virus^7^. According to studies in India, elimination of Piper yellow mottle virus (PYMoV) increased when cyclic somatic embryos were pretreated with ribavirin before induction^46^.

## Conclusion

Among thermotherapy treatments for virus eradication, storage of plantlets 8 h at 27 °C and 16 h at 38 °C for 18 days was the best treatment considering the highest regeneration percentage of survived meristems. The hormonal combination containing 0.4 mg/L BAP was the best treatment used for establishment of meristem. Plantlets obtained from 0.5 mm meristems, which underwent thermotherapy treatment for 18 days at 8 °C and 16 h at 38 °C (TH1), showed the highest percentage of virus elimination for all three viruses (ACLS, ApM and TRS) in different cultivars.

Combining three methods of thermotherapy (TH1), meristem culture and somatic embryogenesis induction from meristem on MS medium supplemented with 0.5 mg/L 2,4-D and 1 mg/L TDZ is the most effective technique for virus eradication without meristem size challenges.

Virus-free explants were amplified in temporary immersion bioreactor system supplemented with 1 mg/L BAP, 0.5 mg/L GA3, 0.01 mg/L IBA and 3% sucrose and then rooted in the half strength MS (1/2MS) medium supplemented with 1 mg/L IBA and 0.5 mg/L IAA.

## Supplementary Information

Supplementary Information.

## Data Availability

All data generated or analyzed during this study are included in this published article [and its supplementary information files].
