# Supplementary material for: Recovery of virus-free Almond (Prunus dulcis) cultivars by somatic embryogenesis from meristem undergone thermotherapy
Source: Sci Rep. 2022 Sep 2;12:14948. doi: 10.1038/s41598-022-19269-3 (PMC9440082; doi:10.1038/s41598-022-19269-3)
Supplement: Supplementary file 1 — Supplementary Information. [file 41598_2022_19269_MOESM1_ESM.pdf]

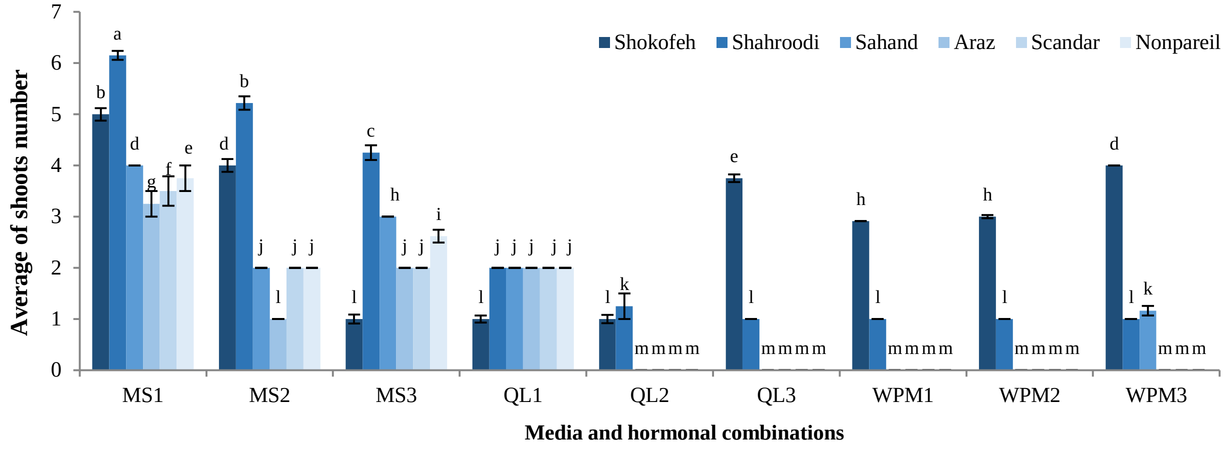

a

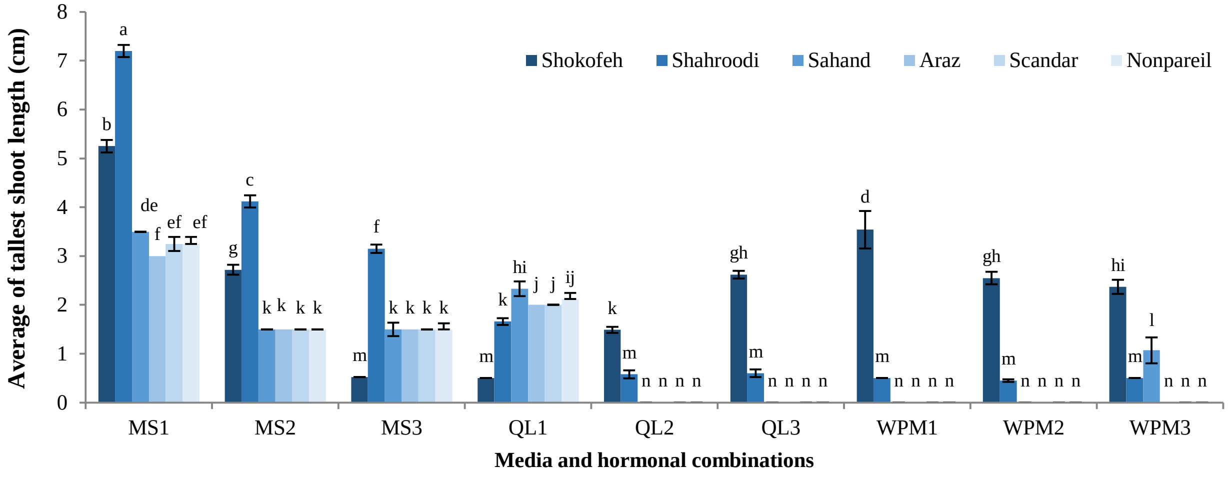

b

Figure 1. sup. Interaction effects of 6 cultivars × 3 media × 3 hormonal combinations on average of shoot number (**a**) and average of tallest shoot length (**b**) after 4 weeks. The figures 1, 2 and 3 on the side of each medium mean the hormonal combination 1, 2 and 3; 1) 1 mg/L BAP + 0.01 mg/L IBA + 0.5 mg/L GA_3, 2) 1 mg/L BAP + 0.01 mg/L IBA and 3) 0.01mg/L IBA + 0.5 mg/L BAP + 0.5 mg/L thidiazuron (TDZ). (n=4, p<0.01).

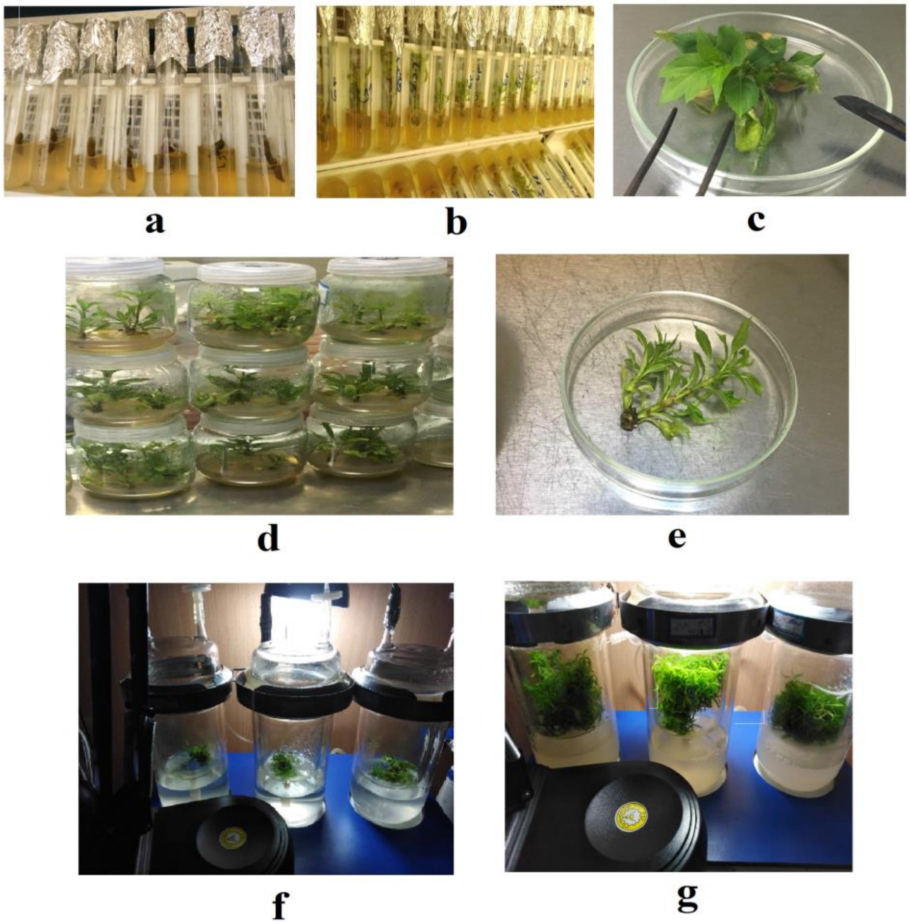

Figure 2. sup. Stages of establishment and micropropagation of almond explants. (**a**) Establishment of explants (**b**) Transfer and growth in growth chambers (**c**) Subculture (**d**) Transfer to solid medium (**e**) Growth in solid medium (**f**) Transfer to temporary immersion bioreactor system (**g**) Grow in temporary immersion bioreactor system.

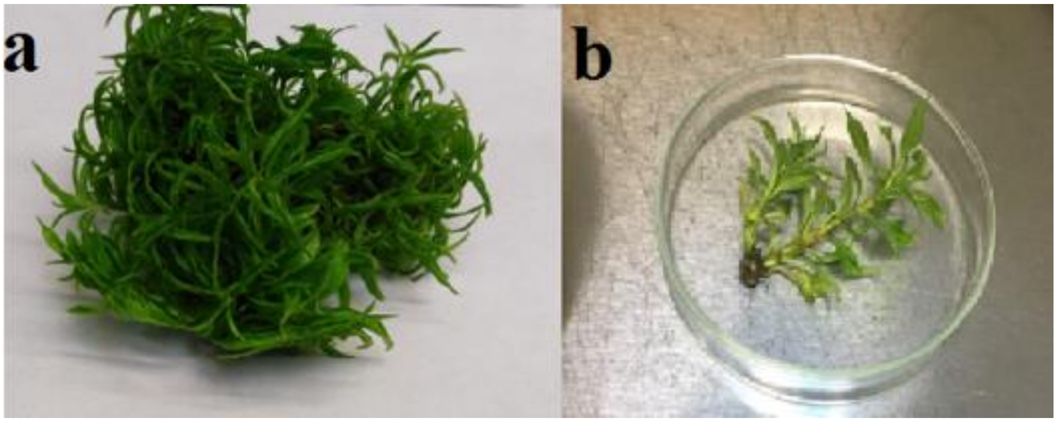

Figure 3. sup. Comparison of solid medium and temporary immersion bioreactor system: (**a**) plantlet mass grown in temporary immersion bioreactor system, (**b**) plantlet grown in solid medium. It should be noted that both plantlet masses are from the proliferation of a nodal explant.

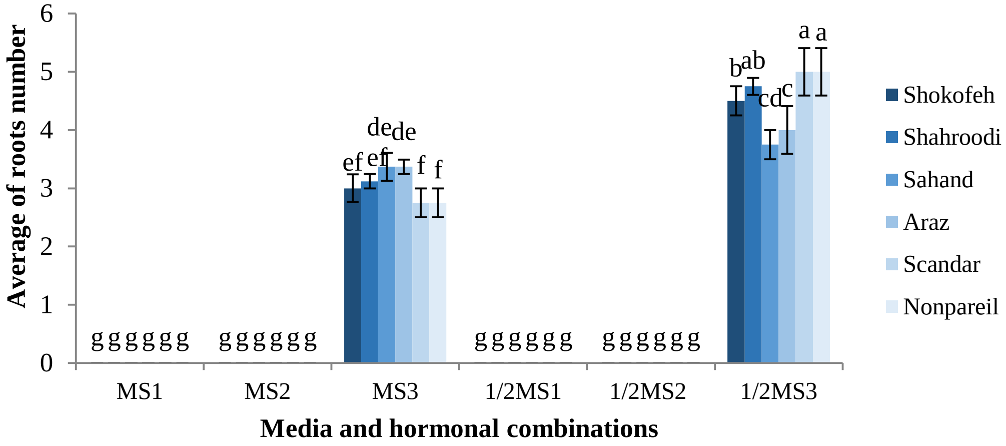

a

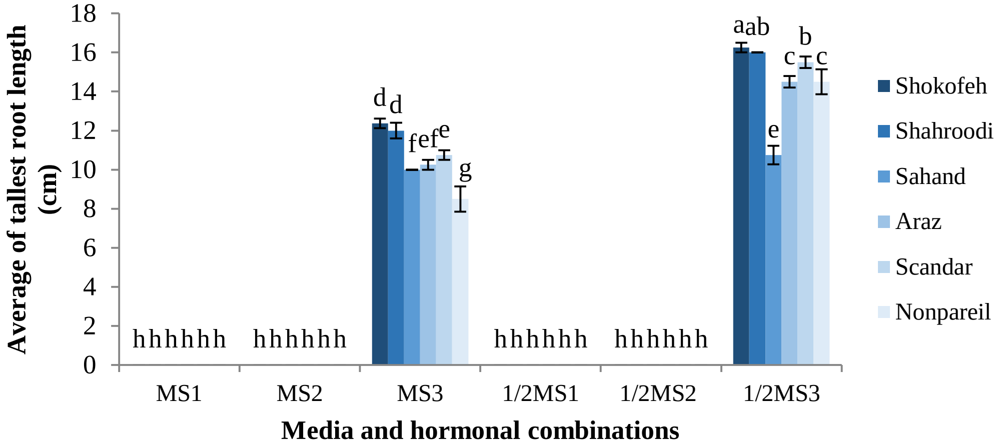

b

Figure 4. sup. Interaction effects of 6 cultivars × 2 media × 3 hormonal combinations on average of root number (**a**) and tallest root length (**b**). The figures 1, 2 and 3 on the side of each medium mean the hormonal combination 1, 2 and 3; 1) Plant growth regulator free media, 2) IAA (1 mg/L) and 3) IAA (1 mg/L) + IBA (0.5 mg/L). (n=4, $p<0.01$).

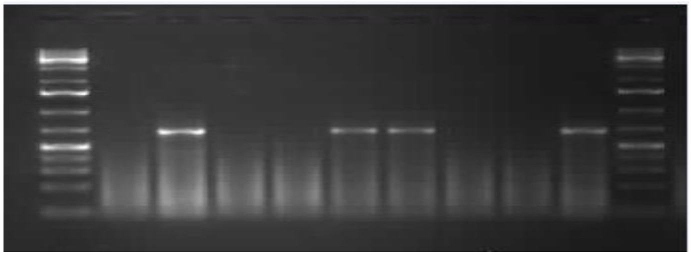

a

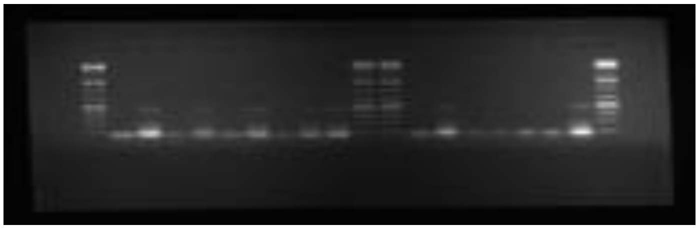

b

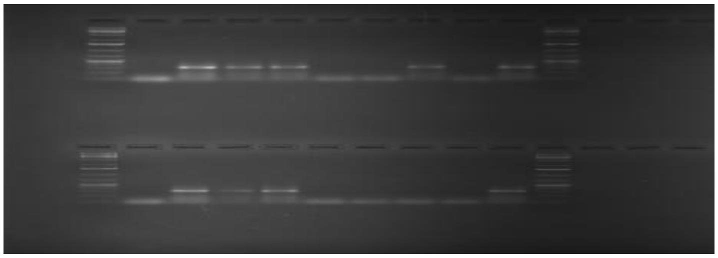

c

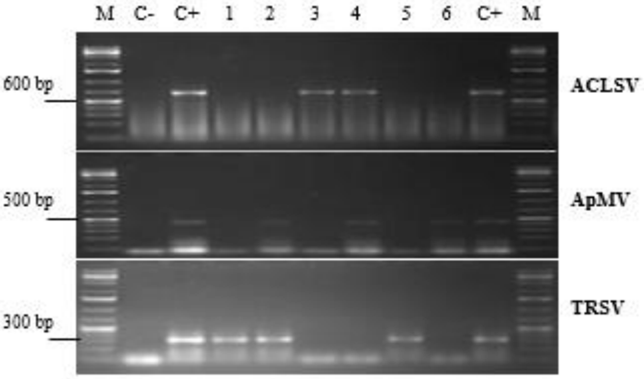

d

Figure 5. sup. Original electrophoretic photos related to identification of ACLSV (**a**), ApMV (**b**) and TRSV (**c**). PCR of mother plants with the primers of ACLS, ApM and TRS viruses (M: 1kb lader, 1- Araz, 2- Sahand, 3- Shahroodi, 4- Shokofeh, 5- Scandar and 6- Nonpareil) (**d**).

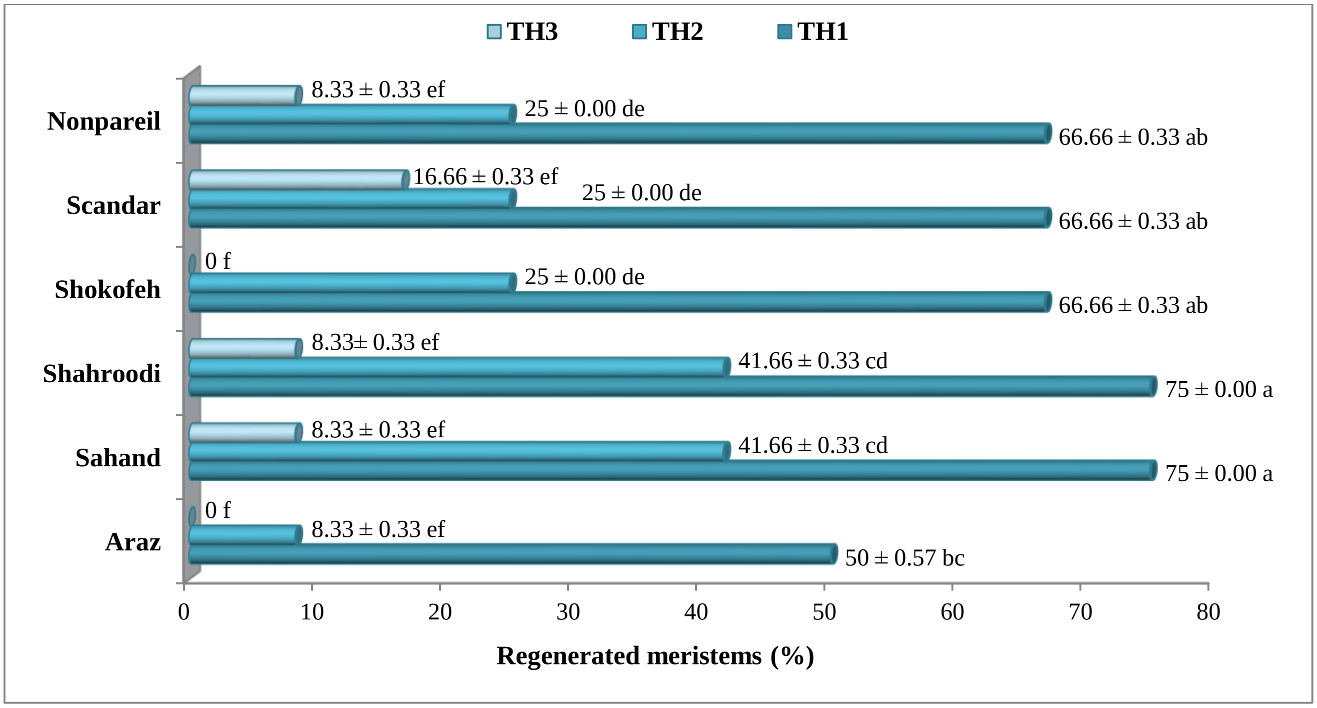

Figure 6. sup. Interaction effects of 6 cultivars × 3 thermotherapy treatments on the percentage of regenerated meristems; TH1: 18 days at 27℃ for 8 h and 38℃ for 16 h, TH2: 10 days at 38℃ and TH3: 11 days at 38℃. (n=3, p<0.05, ± S.E).

Table 1. sup. Interaction effects of 6 cultivars × 2 sizes of meristem × 3 hormonal combinations on the percentage of regenerated meristems. (n=4, p<0.01, ± S.E).

Meristems size (mm)	BAP concentrations (mg/L)	cultivars					
		Shahroodi	Shokofeh	Sahand	Araz	Nonpareil	Scandar
0.5	0.1	0 d	0 d	0 d	0 d	0 d	20 ± 0.20 c
	0.2	0 d	60 ± 0.40 b	60 ± 0.40 b	20 ±0.20 c	0 d	20 ± 0.20 c
	4.0	20 ± 0.17 c	60 ± 0.40 b	60 ± 0.40 b	60 ± 0.40 b	0 d	60 ± 0.40 b
1	0.1	0 d	0 d	0 d	0 d	20 ± 0.20 c	20 ± 0.20 c
	0.2	0 d	60 ± 0.40 b	60 ± 0.40 b	60 ± 0.40 b	20 ± 0.20 c	20 ± 0.20 c
	4.0	60 ± 0.40 b	60 ± 0.40 b	60 ± 0.40 b	80 ± 0.06 a	20 ± 0.20 c	60 ± 0.40 b

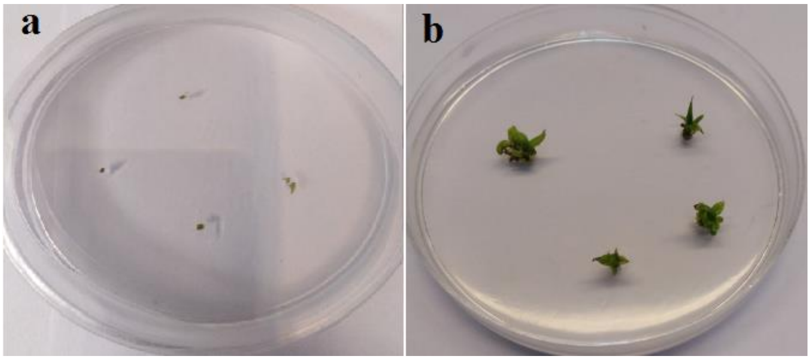

Figure 7. sup. Meristem cultured from Araz cultivar in MS medium containing 0.4 mg/L BAP and 0.1 mg/L IBA. a) Meristems immediately after culture on the medium. b) Meristems after four weeks of culture on the medium.

Table 2. sup. Interaction effects of each cultivar × 2 sizes of meristem × 2 thermotherapy treatments on the percentage of virus elimination. (n=3, p<0.05).

	**meristem size (mm)**	**Viruses**	**Virus-free plantlets (%)**					
			**Shokofeh**	**Shahroodi**	**Sahand**	**Nonpareil**	**Araz**	**Scandar**
**Thermotherapy 1**	**0.5**	**ACLS**	78 a	76.66 a	-	-	-	-
		**ApM**	68.33 a	-	65 a	75 a	-	-
		**TRS**	-	-	62.66 a	-	61.66 a	75 a
	**1**	**ACLS**	54.66 b	54 b	-	-	-	-
		**ApM**	43.66 b	-	46 b	49.66 b	-	-
		**TRS**	-	-	61.33 a	-	58.33 a	64.33 b
**Thermotherapy 2**	**0.5**	**ACLS**	52.66 b	51.33 b	-	-	-	-
		**ApM**	30.33 c	-	38 c	42.66 b	-	-
		**TRS**	-	-	35 b	-	27.33 b	61.66 b
	**1**	**ACLS**	25.33 c	26.33 c	-	-	-	-
		**ApM**	21 d	-	22 d	33.66 c	-	-
		**TRS**	-	-	23.66 c	-	18.66 c	34.66 c

Table 3. sup. Comparison of the results obtained by ELISA and RT-PCR.

		**Almond Cultivars**					
		Araz	Sahand	Shahroodi	Shokofeh	Scandar	Nonpareil
**ELISA result**	Rep. 1	3.309	4.401	0.051	0.046	0.129	0.063
**ELISA result**	Rep. 2	3.208	3.931	0.069	0.039	0.134	0.059
**ELISA result**	Rep. 3	3.273	3.769	0.057	0.042	0.154	0.051
**TRSV C+**		3	3	3	3	3	3
**TRSV C-**		0.08	0.08	0.08	0.08	0.08	0.08
**RT-PCR result**		+	+	-	-	+	-

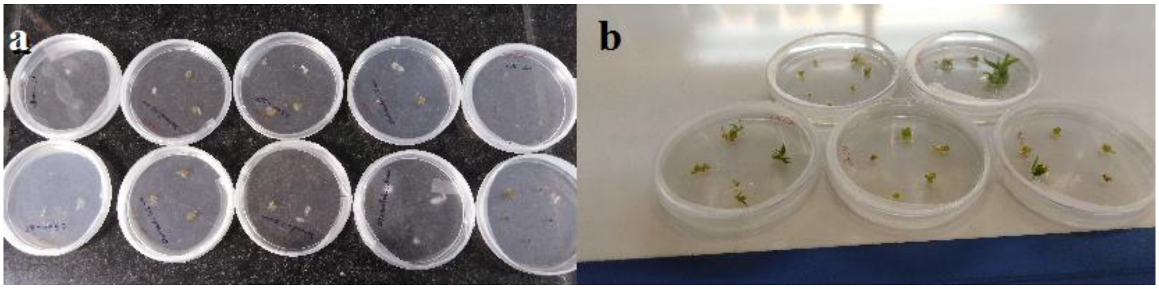

Figure 8. sup. Induction of somatic embryogenesis from meristem undergone thermotherapy (after about 30 days): TH2 led to the production of embryogenic calluses but no plantlets were obtained (**a**), TH1 treatment produced embryogenic calluses and plantlets (**b**).
